# Effect of socioeconomic disparities on the risk of COVID-19 in 8 metropolitan cities in the Korea: a community-based study

**DOI:** 10.4178/epih.e2022107

**Published:** 2022-11-15

**Authors:** Myung-Jae Hwang, Shin Young Park, Tae-Ho Yoon, Jinhwa Jang, Seon-Young Lee, Myeongsu Yoo, Yoo-Yeon Kim, Hae-Kwan Cheong, Donghyok Kwon, Jong-Hun Kim

**Affiliations:** 1 Division of Public Health Emergency Response Research, Korea Disease Control and Prevention Agency, Cheongju, Korea; 2 Department of Preventive Medicine, Pusan National University School of Medicine, Busan, Korea; 3 Department of Social and Preventive Medicine, Sungkyunkwan University School of Medicine, Suwon, Korea

**Keywords:** COVID-19, Socioeconomic disparities in health, Area deprivation index, Community, Korea

## Abstract

**OBJECTIVES:**

Socioeconomic disparities have been reported as major risk factors contributing to the spread of coronavirus disease 2019 (COVID-19) at the community level. We conducted an epidemiological study on COVID-19 incidence risk using area-based deprivation indices (DIs) reflecting the characteristics of the susceptible population.

**METHODS:**

A database of the confirmed COVID-19 cases in 8 metropolitan cities in Korea from January 20, 2020 to December 31, 2021 was combined with area-based DI scores and standardized prevalence rates of diabetes and hypertension from the Korean Community Health Survey. Relative risk (RR) levels were estimated using a generalized linear model with a Poisson distribution by age group.

**RESULTS:**

The risk of COVID-19 incidence generally increased with increasing age, especially in patients aged ≥75 years. The RR of COVID-19 incidence per interquartile range increment of the composite deprivation index (composite DI) was 1.54 (95% confidence interval [CI], 1.34 to 1.70). Notably, in the first wave, the risk of COVID-19 incidence was approximately 3 times higher in the region with the lowest socioeconomic status than in the region with the highest status (RR, 3.08; 95% CI, 2.42 to 3.78 based on the the composite DI and RR, 3.13; 95% CI, 2.53 to 3.83 based on the social deprivation index).

**CONCLUSIONS:**

This study provides scientific evidence that socioeconomic deprivation is an important risk factor for the spread of COVID-19. This finding suggests that a mid-term to long-term strategy is needed to protect susceptible populations and reduce the burden of COVID-19 in the community.

## GRAPHICAL ABSTRACT

[Fig f4-epih-44-e2022107]

## INTRODUCTION

Since the first patient with coronavirus disease 2019 (COVID-19) was confirmed in Korea on January 20, 2020, the number of cases has surpassed 10 million (as of April 2022), leading to a strong sense of crisis. To prevent the spread of COVID-19, governments have implemented guidelines such as hand washing, social distancing, and quarantine. However, reported difficulty exists in the practical application of these guidelines in residential areas with extremely poor living conditions [1]. In the early stages of the COVID-19 pandemic, the virus spread mainly in countries with high economic status, such as the United States, France, Spain, and Korea; thus, the government recommendations to control the spread of infection were predicated on the availability of basic infrastructure, such as sanitary and medical facilities. These guidelines were derived under the premise of the highest living standards [2], including claims made by international organizations [3]. Overcrowded neighborhoods, lack of drinkable water, poor sanitary conditions in the home, lack of awareness of the importance of personal hygiene, and insufficient vaccination were found to be among the key risk factors in the pandemic [4]. Daily workers with unstable economic lives can struggle to maintain a livelihood because of home quarantine; additionally, even if they stay at home, the lack of in-house sanitation facilities may leave them no choice but to use public facilities near their residences [2]. Additionally, people living in extremely poor housing conditions often have underlying or chronic diseases and limited access to medical facilities, leading to serious complications when infected with severe acute respiratory syndrome coronavirus 2 (SARS-CoV-2) [2]. Such health-related inequities may create disparities among regions at different socioeconomic levels within a country [5].

As the COVID-19 pandemic continues worldwide, concerns are growing about areas with large populations, poor health, and low socioeconomic status. Overcrowded neighborhoods and residential environments, lack of basic living infrastructure, informal or irregular work, lack of housing stability, and the need to use public facilities hinder the practice of social distancing and basic hygiene, thus increasing the risk of the spread of COVID-19 [6-9]. However, setting practical policies for areas vulnerable to infection is challenging due to a lack of reliable data on the political, economic, and social contexts that characterize the specific environment of each region [1,2].

Therefore, we examined the risk of COVID-19 incidence by regional-level socioeconomic deprivation and presented scientific evidence that can be used to establish a regional health plan as part of a mid-term to long-term strategy for epidemic control in 8 metropolitan cities in Korea.

## MATERIALS AND METHODS

### Study participants

This study was conducted based on the Korean national database of confirmed patients with COVID-19 reported through the integrated National Notifiable Disease Surveillance System in the epidemiological investigation conducted by the KDCA from January 20, 2020 to December 31, 2021. The participants in our study included those ≥ 20 years old who were reported in Seoul, Busan, Daegu, Incheon, Gwangju, Daejeon, Ulsan, and Sejong as cases of community infection (excluding patients infected abroad or reported in Gyeonggi-do, Gangwon-do, Chungcheongbuk-do, Chungcheongnam-do, Jeollabuk-do, Jeollanam-do, Gyeongsangbuk-do, Gyeongsangnam-do, and Jeju-do). During the study period, the total number of confirmed COVID-19 cases was 279,825.

### Data sources

An area deprivation index (DI) is an indicator of regional health and socioeconomic inequality [10]. For the area DI analysis, we obtained a 10% sample of survey data from Statistics Korea in 2015. DIs were calculated by summing z-standardized scores to attain a population-weighted mean of 0 and a variance of 1. Individual area-based DIs were measured based on the proportions of poor residential environments, households with no car, low educational attainment, aging population, low social class, non-apartment households, single-occupant households, female heads of household, and divorced or separated people by city, county, or district (*si-gun-gu*) [10]. These variables are defined in Supplementary Material 1. After normality testing for these constituent indicators, if the normal distribution did not apply, factor analysis was performed through transformation [11]. We used the composite deprivation index (composite DI), economic deprivation index (economic DI), social deprivation index (social DI), and DI of factors related to mortality (mortality-related DI) as indices for socioeconomic disparities at the regional level in 2015.

In this study, Korean Community Health Survey (CHS) data were used to assess health vulnerability at the community level. The CHS is a national survey of 253 administrative districts (*si-gun-gu*) comprising all residents aged ≥ 19 years, obtained through primary (sample location) and secondary (sample household) probability sampling [12]. We used the regional standardized prevalence rates of diabetes and hypertension from the 2020 CHS survey as indices of health vulnerability. Furthermore, data regarding the COVID-19 vaccination rate were obtained from the integrated system in an epidemiological investigation by the KDCA. Vaccination began in Korea in February 2021. Since the vaccination rate continues to change, we calculated the first, second, and third vaccination rates on November 1 and December 23, 2021, respectively, after the start of the third vaccination.

### Definitions of covariates

The COVID-19 incidence per 100,000 population was calculated based on the resident registration population status data provided by the Ministry of Public Administration and Security as of December 2020. The fatality rate was calculated as the number of COVID-19 deaths divided by the number of confirmed cases, multiplied by 100. The COVID-19 incidence and fatality rates were estimated by region at the *si-gun-gu* level.

In this study, we used the composite area DI for Korea [10]. The composite DI was calculated using the following indicators: poor residential environments, no-car households, low educational attainment, aging population, low social class, non-apartment households, single-occupant households, female heads of household, and divorced or separated people. The economic DI was calculated based on rates of non-apartment households, poor residential environments, low educational attainment, aging population, and low social class. The social DI was calculated based on rates of single-occupant households, no-car households, nonapartment households, female heads of household, and divorced or separated people. The mortality-related DI was calculated using the indicators most strongly correlated with mortality: no-car households, low educational attainment, low social class, and divorced or separated people. These indicators were selected based on significant results from factor analysis, and the DI was calculated as the sum of the standardized scores [11]. Generally, a high value indicates an area with a large proportion of aging population, low educational attainment, and poor residential environments, whereas a lower value indicates the opposite [10].

Indices of the regional prevalence levels of diabetes and hypertension were defined as the standardized prevalence of participants aged > 19 years diagnosed with the disease by a doctor. Furthermore, age standardization was applied for comparison with indicators surveyed in other regions or at other time points, as the age-specific population structure that impacts health status differs depending on the survey region and time point [12].

We associated the COVID-19 incidence and fatality rates reported by *si-gun-gu* with scores on several area-based DIs (composite DI, economic DI, social DI, and mortality-related DI) as well as the standardized prevalence rates of diabetes and hypertension.

### Study design

We calculated the risks of COVID-19 incidence and fatality according to the regional-level area-based DIs throughout the study period. Then, we estimated the risk by age group, with participants divided into those aged 20-39 years, 40-59 years, 60-74 years, and ≥ 75 years. The COVID-19 incidence risk was evaluated for each epidemic period, divided into the first wave (from February 18 to May 5, 2020), the second wave (from August 12 to November 12, 2020), the third wave (from November 13, 2020 to January 20, 2021), and the fourth wave (from July 7 to December 31, 2021) in Korea. In patients aged ≥ 75 years, the risk of COVID-19 incidence was estimated for the 4 quantiles of the composite DI and the social DI for each epidemic period. For composite DI, quantile 1 (Q1, reference) included areas with scores less than -7.9, quantile 2 (Q2) included those with scores greater than -7.9 and less than -4.8, quantile 3 (Q3) represented scores greater than -4.8 and less than -2.1, and quantile 4 (Q4) represented scores of 2.1 or more. As for social DI, Q1 (reference) included regions with scores less than -3.7, Q2 included those with scores greater than -3.7 and less than -1.3, Q3 represented scores greater than -1.3 and less than 1.3, and Q4 represented scores ≥ 1.3. Additionally, we analyzed the rate of SARS-CoV-2 vaccination by quantile of the composite DI at the regional level.

### Statistical analysis

We carried out a retrospective cohort study using a generalized linear model with a Poisson distribution to observe the risk of SARS-CoV-2 infection per interquartile range (IQR) increment of each area-based DI, adjusting for the standardized prevalence levels of diabetes and hypertension. To determine the optimal model, the Akaike criterion was calculated for each model (Supplementary Material 2). All statistical analyses were performed using R version 4.0.4 (R Foundation for Statistical Computing, Vienna, Austria) software.

### Ethics statement

This study was approved by the Institutional Review Board of the Korea Disease Control and Prevention Agency (KDCA; approval No. KDCA 2021-04–07-PE-A). The board waived the requirement to obtain informed consent.

## RESULTS

The confirmed COVID-19 cases included 139,585 males and 140,240 females, for a total of 279,825 cases (Table 1). These were divided into age groups, with 97,513 (34.8%) aged 20-39 years, 96,431 (34.5%) aged 40-59 years, 66,586 (23.8%) aged 60-74 years, and 19,295 (6.9%) aged ≥ 75 years. Based on the regional distribution, the number of confirmed cases reported in Seoul was 67.5%, accounting for more than half of the total cases. During the study period, 3,289 people died due to COVID-19, with a fatality rate of 1.2%.

After calculating the area-based DI scores, the mean composite DI was determined to be -4.7. The highest composite DI was in Jung-gu (6.5), Busan and the lowest in Yuseong-gu (-13.9), Daejeon. The mean economic DI was -3.6, with the highest in Ganghwa-gun (4.2), Incheon and the lowest in Gangnam-gu (-9.7), Seoul. The mean social DI was -1.1, with the highest in Jung-gu (8.7), Busan and the lowest in Buk-gu (-11.1), Ulsan. The mean mortality-related DI was -1.9, with the highest in Jung-gu (3.0), Busan and the lowest in Seocho-gu (-8.5), Seoul. The mean standardized diabetes prevalence rate was 8.2%, with the highest prevalence in Jungnang-gu (11.3%), Seoul and the lowest in Yuseonggu (5.5%), Daejeon (Table 2). Finally, the mean standardized hypertension prevalence rate was 18.6%, with the highest prevalence in Jung-gu (23.9%), Incheon and the lowest in Nam-gu (14.8%), Gwangju.

**Figure 1.**
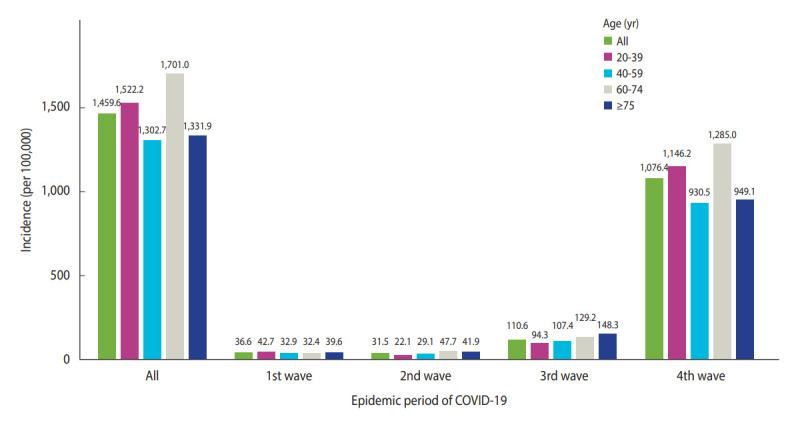
Coronavirus disease 2019 (COVID-19) incidence by age group during the entire study period and each epidemic period.

The COVID-19 incidence rate was calculated based on age group and epidemic period (Figure 1). During the study period, the total incidence per 100,000 population was 1,459.6 among all participants and 1,701.0, 1,522.2, 1,331.9, and 1,302.7 among those aged 60-74 years, 20-39 years, ≥ 75 years, and 40-59 years, respectively. The COVID-19 incidence rates per 100,000 population in the first, second, third, and fourth waves were 42.7 (with the highest incidence in the age group of 20-39 years), 47.7 (with the highest incidence in the age group of 60-74 years), 148.3 (with the highest incidence in the age group of ≥ 75 years), and 1,285.0 (with the highest incidence in the age group of 60-74 years), respectively. This result was supported by clear variation in the COVID-19 outbreak pattern and age group of spread by epidemic period. The COVID-19 incidence rates at the *si-gun-gu* level during the study period and each epidemic period are shown in Supplementary Material 3.

Based on generalized linear modeling of the risk of COVID-19 incidence associated with the area-based DIs, a statistically significant result was observed in model 3, which was the optimal model in our study (Table 3). A strong correlation was noted between the risk of COVID-19 incidence and the composite DI. The relative risk (RR) per IQR increment was higher for the composite DI (1.44; 95% confidence interval [CI],1.35 to 1.57) than for the other area-based DIs. Furthermore, the RRs per IQR increment for the economic DI (1.05; 95% CI, 1.02 to 1.07) and the social DI (1.10; 95% CI, 1.01 to 1.19) constituted significant findings. However, the RR per IQR increment for mortality-related DI (0.97; 95% CI, 0.90 to 1.04) did not constitute a significant result.

Overall, the association between the risk of COVID-19 incidence and the IQR increment of the area-based DIs varied significantly by age group (excluding mortality-related DI), with the strongest relationship found among those aged ≥ 75 years (Table 4). In that age group, the RR of incidence per IQR increment was 1.54 (95% CI, 1.34 to 1.70) for the composite DI, 1.06 (95% CI, 1.02 to 1.10) for the economic DI, and 1.21 (95% CI, 1.16 to 1.27) for the social DI.

**Figure 2.**
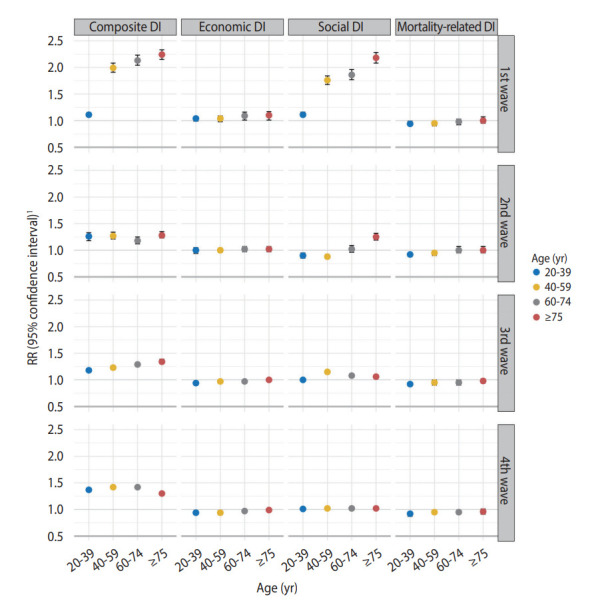
Relative risk (RR) of coronavirus disease 2019 incidence per interquartile range increment of area deprivation index scores by epidemic period and age group in model 3. Composite DI, composite deprivation index; Economic DI, economic deprivation index; Social DI, social deprivation index; Mortality-related DI, deprivation index of factors related to mortality. ^1^RR was estimated by adjusting for the level of the standardized prevalence of hypertension and diabetes.

Based on these results, the risk of SARS-CoV-2 infection by age group was analyzed according to the epidemic period (Figure 2). Here, the relationship between COVID-19 risk and area-based DI score also strengthened with increasing age. This effect was most conspicuous during the first wave, with an obvious pattern. In the first wave, the RRs per IQR increment for composite DI and social DI were 2.24 (95% CI, 2.15 to 2.33) and 2.18 (95% CI, 2.08 to 2.28), respectively, in patients aged ≥ 75 years. In the second wave, the RRs per IQR increment for composite DI and social DI were 1.28 (95% CI, 1.23 to 1.35) and 1.25 (95% CI, 1.19 to 1.32), respectively, in that age group. During the third wave of the epidemic, this pattern showed similar results for composite DI. In the third wave, the RR per IQR increment for composite DI was 1.34 (95% CI, 1.30 to 1.39) for those aged ≥ 75 years, among whom it was highest; however, consistent results were not observed during the fourth wave. Moreover, no significant relationship with COVID-19 incidence according to age group was found for economic DI or mortality-related DI.

**Figure 3.**
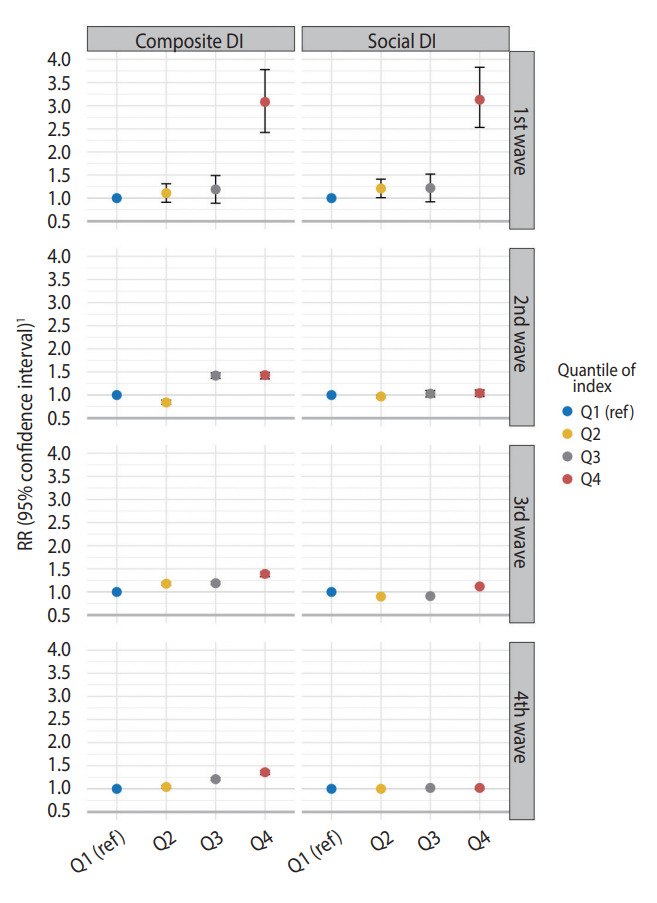
Relative risk (RR) of coronavirus disease 2019 incidence associated with the 4 quantiles of composite deprivation and social deprivation index scores in those ≥75 years old by epidemic period. Composite DI, composite deprivation index; Social DI, social deprivation index; ref, reference value. ^1^RR was estimated by adjusting for the standardized prevalence of hypertension and diabetes.

Based on previous results, the risk of COVID-19 incidence was estimated by dividing the composite DI and social DI scores into quartiles by region in patients aged ≥ 75 years, which showed significant results (Figure 3). As the quantiles of composite DI and social DI increased, the risk of COVID-19 increased. During the first wave, the COVID-19 risk for the areas in Q4 by the composite DI was 3.08 (95% CI, 2.42 to 3.78) and the risk for those in Q4 by the social DI was 3.13 (95% CI, 2.53 to 3.83) times higher than the risk in the Q1 areas for the respective measure. In the areas in Q4 of the composite DI, the RRs for the second, third, and fourth waves were 1.43 (95% CI, 1.35 to 1.49), 1.39 (95% CI, 1.33 to 1.43), and 1.36 (95% CI, 1.31 to 1.40), respectively. In contrast, considering social DI quantiles, the relationship with COVID-19 risk was significant in only the first (3.13; 95% CI, 2.53 to 3.83).and third waves (1.12; 95% CI, 1.10 to 1.14).

## DISCUSSION

We explored the effects of socioeconomic disparities on the risk of COVID-19 at the regional level. Our findings indicated that the risk of COVID-19 incidence and fatality increased with regional socioeconomic deprivation (Supplementary Material 4). Furthermore, greater sensitivity to this relationship was observed among patients aged ≥ 75 years. Additionally, a greater risk of COVID-19 was reported in areas with higher quantiles of composite DI and social DI. To our knowledge, based on the determinants of the spread of infection in each area, the COVID-19 risk level differed according to the epidemic period. The pattern of increased COVID-19 incidence risk with an increasing level of socioeconomic deprivation was most prominent during the early stages of the COVID-19 epidemic (i.e., the first and second waves) in Korea. This is attributable to the lack of information and awareness regarding the risk and prevention of SARS-CoV-2 infection in the early stages of the epidemic. In particular, the risk would be expected to be higher in the older population, who may have limited access to information from the media. Low socioeconomic levels, poor access to health care, and occupational and environmental factors may also increase potential exposure to viruses at the community level [2,4]. Thus, we observed that these socioeconomic factors may serve as determinants for the transmission of infectious diseases.

An area-based DI is a representative indicator of health determinants, such as poor residential environment, public health susceptibility, and poor access to sanitation and health facilities at the community level [12]. People living in poor housing conditions can experience serious complications when infected due to difficulty receiving timely and appropriate medical services, leading to a high fatality rate [13]. Moreover, the potentially fatal risk of spreading infectious diseases, such as COVID-19, in low-income areas can generate socioeconomic inconsistencies due to preventive measures and variations in health behavior [14]. Specifically, populations in more socioeconomically deprived areas have a higher risk of infection due to a lack of prior knowledge and prevention of infectious diseases such as COVID-19. The Korean CHS conducted for 2020, when the epidemic began, revealed lower compliance with safety and quarantine rules for the prevention of infectious diseases among those with lower education or income levels [15]. Furthermore, the implementation of preventive measures and quarantine rules differed according to the relative level of inequality [15]. A cohort study based on the Korean National Health Insurance database revealed that the risk of SARS-CoV-2 infection was 22% higher among Medical Aid beneficiaries than others [16].

Additionally, regional socioeconomic disparities have been reported as a determinant of the spread of SARS-CoV-2 infection [17]. KC et al. [18] reported that the risk of COVID-19 was nearly 40% higher in the quantile with the lowest neighborhood socioeconomic status than in that with the highest status, underscoring the association between socioeconomic status and access to healthcare services. In a similar study, for 7 states in the United States (Arizona, Florida, Illinois, Maryland, North Carolina, South Carolina, and Virginia), a strong relationship was reported between a composite area DI and COVID-19 incidence [19]. A comparison by income in the New York metropolitan area revealed that the infection rate was lower in zip codes with higher income levels [20]. Moreover, socioeconomically disadvantaged people have been shown to have an increased risk of COVID-19 mortality, with a mortality rate 3.2 times higher in migrants than in general populations [2]. Our study results were consistent with those of previous studies.

These socioeconomic disparities between regions were associated with the prevalence rates of diabetes and hypertension, which are common chronic diseases [21]. Such gaps are a likely contributor to regional health inequality. In our study, similar to previous studies, an analysis of the risk of COVID-19 incidence and fatality according to the regional standardized prevalence of diabetes and hypertension showed that the risk of infection increased with increasing morbidity [22,23]. Furthermore, the observed COVID-19 risk among patients with diabetes and hypertension is likely multifactorial. According to previous reports, diabetes may promote the entry of SARS-CoV-2 via the increased expression of angiotensin-converting enzyme 2 surface receptors, due both to the disease itself and the therapeutic strategies used. Moreover, cytokine storms and end-organ damage may be major contributors to this risk due to the underlying immune dysregulation, which can tend to promote exaggerated immune responses to viral exposure in patients with hypertension [24,25]. Hence, in the present study, we estimated the risk of COVID-19 incidence by adjusting for the regional prevalence of diabetes and hypertension in 2020, when the epidemic started, and epidemiologically demonstrated the risk of COVID-19. We also observed that the risk of COVID-19 incidence is significantly associated with the regional level of diabetes and hypertension (Supplementary Materials 5 and 6).

However, our study had some limitations. First, COVID-19 incidence was estimated based on the reported address of the reporting medical institution, not the residential address of the patient. Epidemiological information on COVID-19 is entered into the National Notifiable Disease Surveillance System by the KDCA, and the residential addresses of confirmed patients are self-reported, thus leading to missing data in the national database. As such, if SARS-CoV-2 infection testing is performed somewhere other than the patient’s region of residence, a bias may be present between the residential and reported addresses. Second, the study was conducted only in metropolitan cities and not in all regions of the Korea. In rural areas with a low population density, the incidence of SARS-CoV-2 infection was low during the study period and showed no clear relationship with socioeconomic DIs. As such, although not nationwide, a significant difference in risk level was observed according to regional socioeconomic disparities within large cities [19]. Although this has the advantage of reducing the variation in population density, which is an important risk factor for infectious diseases (such as SARS-CoV-2) transmitted through contact with confirmed patients, limitations exist in generalizing the study results on a national level. Third, demographic characteristics such as sex, non-pharmacological interventions such as social distancing, and factors attributable to the risk of spreading infectious diseases, such as vaccination rate, were not considered [26]. Additionally, we observed the composite DI and COVID-19 vaccination rate at the regional level according to the timing of vaccination in Korea (Supplementary Materials 7 and 8). No significant difference in the COVID-19 vaccination rate was noted at each time point, with the vaccine being preferentially administered to essential medical personnel, those aged ≥ 75 years, and residents in the area with the highest quantile of the composite DI in 2021. Finally, given the use of the available area DI data from 2015, relatively recent information was not reflected in our study. The area DI is an indicator calculated once every 5 years. Hence, if our study had been conducted based on the 2020 area DI, we could have evaluated the risk of COVID-19 incidence more accurately by the level of health and the socioeconomic gap between regional levels.

Despite these limitations, this study has the strength of evaluating the risk of COVID-19 incidence and fatality using area-based DIs at the *si-gun-gu* level in Korea. Additionally, the risk of incidence by age group was calculated according to the epidemic period, and the risk of SARS-CoV-2 infection was found to be higher in the more socioeconomically vulnerable population. Therefore, our study underscores the necessity of reducing the burden of COVID-19, identifying susceptible populations at the local level, establishing health improvement measures such as improving access to medical services, and achieving the effects of the guidelines recommended by the government. This suggests that socioeconomic support is needed for susceptible populations [2], such as the unemployed or extremely impoverished, who have trouble maintaining a stable livelihood, and that quarantine products should be distributed to those living in poor environments. Since accurate information is needed to prevent the spread of SARS-CoV-2 infection in advance, efforts should be made to deliver this information quickly and accurately to those with limited access to the media or news. Based on this study, with the ongoing spread of SARS-CoV-2 infection, each local government should implement continuous and appropriate preventive measures to support susceptible populations. We also suggest the need for a mid- to long-term strategy regarding socioeconomic inclusion.

In conclusion, during the COVID-19 epidemic in Korea from 2020 to 2021, the risk of incidence increased as the level of socioeconomic deprivation in the area increased, especially among older adults. Based on the study results, we recommend the establishment of a mid- to long-term strategy for the sustained protection of susceptible populations in Korea, where the COVID-19 epidemic continues.

## Figures and Tables

**Figure f4-epih-44-e2022107:**
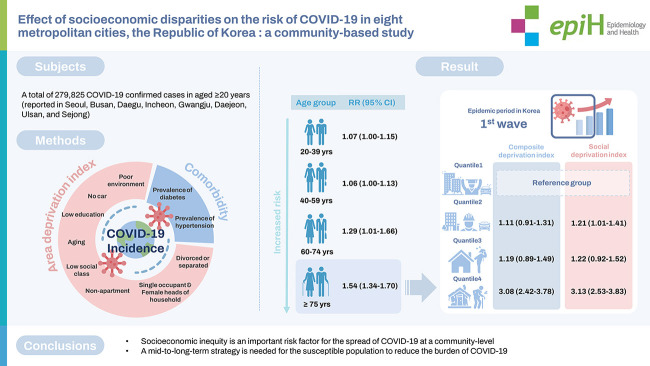


**Table 1.** Demographic characteristics of confirmed COVID-19 cases in 8 metropolitan cities in Korea from January 20, 2020 to December 31, 2021

Characteristics		Male	Female	Total	p-value^1^
Total		139,585 (100)	140,240 (100)	279,825 (100)	<0.05
Age (yr)					<0.05
	20-39	51,588 (37.0)	45,925 (32.7)	97,513 (34.8)	
	40-59	47,771 (34.1)	48,660 (34.7)	96,431 (34.5)	
	60-74	32,054 (23.0)	34,532 (24.6)	66,586 (23.8)	
	≥75	8,172 (5.9)	11,123 (8.0)	19,295 (6.9)	
Administrativedistrict					<0.001
	Seoul (25 districts)	96,057 (68.8)	92,839 (66.2)	188,896 (67.5)	
	Busan (16 districts)	9,622 (6.9)	10,662 (7.6)	20,284 (7.2)	
	Daegu (8 districts)	8,628 (6.2)	10,653 (7.6)	19,281 (6.9)	
	Incheon (9 districts)	14,363 (10.3)	14,530 (10.4)	28,893 (10.3)	
	Gwangju (5 districts)	2,894 (2.1)	3,335 (2.4)	6,229 (2.2)	
	Daejeon (5 districts)	4,533 (3.2)	4,820 (3.4)	9,353 (3.3)	
	Ulsan (5 districts)	2,690 (1.9)	2,715 (1.9)	5,405 (2.0)	
	Sejong (1 district)	798 (0.6)	686 (0.5)	1,484 (0.6)	
Deaths (fatality)		1,719 (1.2)	1,570 (1.1)	3,289 (1.2)	<0.05

Values are presented as number (%). COVID-19, coronavirus disease 2019.

1 Using the chi-square test or the Fisher exact test.

**Table 2.** Distribution of area deprivation indices and health vulnerability

Index		Mean	SD	Min	Percentile			Max	IQR
					25	50	75		
Area deprivation index									
	Composite deprivation index	-4.7	4.6	-13.9	-7.8	-4.8	-2.1	6.5	5.7
	Economic deprivation index	-3.6	2.4	-9.7	-5.1	-3.8	-2.1	4.2	3.0
	Social deprivation index	-1.1	3.6	-11.1	-3.7	-1.3	1.3	8.7	5.0
	Deprivation index of factors related to mortality	-1.9	2.2	-8.5	-2.9	-1.9	-0.6	3.0	2.3
Health vulnerability									
	Standardized prevalence of diabetes (%)	8.2	1.2	5.5	7.2	8.0	9.1	11.3	1.9
	Standardized prevalence of hypertension (%)	18.6	2.2	14.8	17.1	18.1	20.2	23.9	3.1

SD, standard deviation; Min, minimum; Max, maximum; IQR, interquartile range.

**Table 3.** Relative risk of COVID-19 incidence per interquartile range increment of area deprivation indices during the study period^1^

Area deprivation index	Model 1	Model 2	Model 3
Composite deprivation index	1.40 (1.29, 1.54)^*^	1.41 (1.30, 1.53)^*^	1.44 (1.35, 1.57)^*^
Economic deprivation index	1.00 (0.93, 1.08)	1.02 (0.95, 1.11)	1.05 (1.02, 1.07)^*^
Social deprivation index	1.03 (0.98, 1.11)	1.04 (1.03, 1.05)^*^	1.10 (1.01, 1.19)^*^
Deprivation index of factors related to mortality	0.95 (0.89, 1.02)	0.97 (0.91, 1.03)	0.97 (0.90, 1.04)

Values are presented as relative risk (95% confidence interval). COVID-19, coronavirus disease 2019.

1 Model 1: crude model; Model 2: adjusted for the standardized prevalence of hypertension; Model 3: model 2+adjusted for the standardized prevalence of diabetes.

* p<0.05.

**Table 4.** Relative risk^1^ of COVID-19 incidence per interquartile range increment of area deprivation indices by age group during the study period

Area deprivation index	Age (yr)			
	20-39	40-59	60-74	≥75
Composite deprivation index	1.07 (1.00, 1.15)	1.06 (1.00, 1.13)	1.29 (1.01, 1.66)^*^	1.54 (1.34, 1.70)^*^
Economic deprivation index	0.97 (0.94, 1.00)	0.97 (0.94, 1.01)	1.00 (0.98, 1.04)	1.06 (1.02, 1.10)^*^
Social deprivation index	0.97 (0.94, 1.00)	0.98 (0.96, 1.03)	1.11 (1.05, 1.18)^*^	1.21 (1.16, 1.27)^*^
Deprivation index of factors related to mortality	0.94 (0.88, 0.99)	0.95 (0.88, 0.99)	1.00 (0.95, 1.03)	1.00 (0.95, 1.07)

Values are presented as relative risk (95% confidence interval). COVID-19, coronavirus disease 2019.

1 Adjusted for the standardized prevalence of hypertension and diabetes.

* p<0.05.

## SUPPLEMENTARY MATERIALS

Supplementary Material 1. Description of the area deprivation index for Korea

Supplementary Material 2. Estimated the Akaike Information Criterion (AIC) for model selection with covariates

Supplementary Material 3. Incidence of COVID-19 by the epidemic period during the study period at *si-gun-gu*

Supplementary Material 4. Relative risk of COVID-19 fatality per interquartile range increment of the level of area deprivation indices

Supplementary Material 5. Relative risk of COVID-19 incidence per interquartile range increment of the standardized prevalence of diabetes

Supplementary Material 6. Relative risk of COVID-19 incidence per interquartile range increment of the standardized prevalence of hypertension

Supplementary Material 7. Association between four quantiles for composite deprivation index and regional vaccination rate of SARS-CoV-2 in (A) all and (B) ≥75 years old population as the number of vaccination on November 1, 2021. †Vaccination rate was calculated as the number of SARS-CoV-2 vaccination completed subjects divided by the number of participants to SARS-CoV-2 vaccination.

Supplementary Material 8. Association between four quantiles for composite deprivation index and regional vaccination rate of SARS-CoV-2 in (A) all and (B) ≥75 years old population as the number of vaccination on December 23, 2021. †Vaccination rate was calculated as the number of SARS-CoV-2 vaccination completed subjects divided by the number of participants to SARS-CoV-2 vaccination.
