# Supplementary material for: Effect of socioeconomic disparities on the risk of COVID-19 in 8 metropolitan cities in the Korea: a community-based study
Source: Epidemiol Health. 2022 Nov 15;44:e2022107. doi: 10.4178/epih.e2022107 (PMC10185970; doi:10.4178/epih.e2022107)
Supplement: Supplementary Material 1. — Description of the area deprivation index for Korea [file epih-44-e2022107-Supplementary-1.pdf]

Supplementary materials

Supplementary Material 1. Description of the area deprivation index for Korea

Indicator	Definition
Poor residential environments	Percent of households under the minimum housing standard (no separate kitchen, no water supply, no hot water for the bathroom, or no flushing toilet)
No car	Percent of households without a car for commuting
Low education attainment	Percent of individuals with education level below high school graduation among the population of those 30-64 years old
Aging population	Percent of elderly individuals 65 years or older in the total population
Low social class	Percent of all persons in households with a head of the household who is engaged in elementary occupation
Non-apartment	Percent of non-apartment households
Single occupant households	Percent of households living alone
Female heads-of-households	Percent of households with female heads-of-household
The divorced or separated	Percent of divorced or widowed individuals
