# Supplementary material for: Effect of socioeconomic disparities on the risk of COVID-19 in 8 metropolitan cities in the Korea: a community-based study
Source: Epidemiol Health. 2022 Nov 15;44:e2022107. doi: 10.4178/epih.e2022107 (PMC10185970; doi:10.4178/epih.e2022107)
Supplement: Supplementary Material 2. — Estimated the Akaike Information Criterion (AIC) for model selection with covariates [file epih-44-e2022107-Supplementary-2.pdf]

Supplementary materials

Supplementary Material 2. Estimated the Akaike Information Criterion (AIC) for model selection with covariates

Area deprivation index	AIC		
	**Model 1 (Crude model)**	**Model 2**	**Model 3**
Composite deprivation index	1,206.87	1,205.33	1,201.18
Economic deprivation index	1,189.99	1,189.45	1,170.78
Social deprivation index	1,197.07	1,196.17	1,184.42
Deprivation index of factors related to mortality	1,207.02	1,205.13	1,201.15

AIC, Akaike Information Criterion. † Estimated using a generalized linear model. Model 1: crude model; model 2: adjusted with the standardized prevalence of hypertension; and model 3: model 2 + adjusted with the standardized prevalence of diabetes.
