# Supplementary material for: Effect of socioeconomic disparities on the risk of COVID-19 in 8 metropolitan cities in the Korea: a community-based study
Source: Epidemiol Health. 2022 Nov 15;44:e2022107. doi: 10.4178/epih.e2022107 (PMC10185970; doi:10.4178/epih.e2022107)
Supplement: Supplementary Material 3. — Incidence of COVID-19 by the epidemic period during the study period at si-gun-gu [file epih-44-e2022107-Supplementary-3.pdf]

Supplementary materials

Supplementary Material 3. Incidence of COVID-19 by the epidemic period during the study period at *si-gun-gu*

Sido	*Si-gun-gu*	Total	Epidemic period			
			1st	2nd	3rd	4th
**Seoul**	**Total**	2,286.1	4.2	51.8	177.7	1,734.9
	Gangnam-gu	2,809.2	5.4	65.0	163.1	1,962.0
	Gangdong-gu	2,063.9	0.8	41.7	117.8	1,574.3
	Gangbuk-gu	2,083.5	1.1	39.6	128.7	1,638.5
	Gangseo-gu	1,797.6	2.6	39.9	212.7	1,359.5
	Gwanak-gu	2,047.6	6.8	70.9	126.6	1,574.9
	Gwangjin-gu	2,134.3	1.0	42.2	121.2	1,601.7
	Guro-gu	2,854.5	15.3	29.8	184.1	2,361.4
	Geumcheon-gu	2,540.6	4.4	43.5	107.6	2,172.0
	Nowon-gu	1,624.3	3.2	55.9	117.3	1,207.0
	Dobong-gu	1,975.8	1.1	80.1	139.6	1,475.9
	Dongdaemun-gu	3,124.0	7.7	59.3	216.8	2,471.8
	Dongjak-gu	2,223.5	4.7	55.2	185.6	1,657.4
	Mapo-gu	1,735.9	2.2	27.9	157.9	1,316.0
	Seodaemun-gu	1,921.2	5.2	53.7	163.6	1,479.6
	Seocho-gu	2,639.4	4.0	53.5	209.9	1,958.7
	Seongdong-gu	1,985.9	3.2	42.2	156.7	1,428.8
	Seongbuk-gu	1,993.6	2.7	58.7	125.5	1,538.6
	Songpa-gu	2,298.9	3.2	52.3	346.0	1,616.2
	Yangcheon-gu	1,708.9	4.6	35.0	167.3	1,306.3
	Yeongdeungpo-gu	3,082.0	3.6	64.1	186.1	2,473.5
	Yongsan-gu	2,241.0	2.5	50.3	146.8	1,548.3

	Eunpyeong-gu	1,939.5	6.1	48.1	147.0	1,463.3
	Jongno-gu	4,124.8	6.1	81.8	385.2	3,222.2

continued

	Jung-gu	6,387.1	7.2	80.6	386.1	5,037.1
	Jungnang-gu	2,098.7	2.6	55.4	175.7	1,536.2
**Busan**	**Total**	702.5	3.5	12.6	55.4	513.0
	Gangseo-gu	684.0	0.9	2.8	41.5	517.2
	Gumjung-gu	611.1	1.5	5.9	43.6	446.7
	Gijang-gun	545.3	-	4.4	17.4	432.9
	Nam-gu	554.6	0.9	5.7	47.2	406.8
	Dong-gu	1,296.6	5.0	12.6	301.2	827.2
	Dongnae-gu	700.0	11.1	11.5	57.5	516.1
	Busanjin-gu	597.0	2.2	16.9	51.0	432.8
	Buk-gu	533.0	2.1	39.7	41.4	401.4
	Sasang-gu	727.5	2.2	15.1	69.7	545.5
	Saha-gu	533.4	3.0	3.8	37.5	335.2
	Seo-gu	1,322.1	8.4	22.1	39.9	861.1
	Suyeong-gu	716.2	4.5	4.5	59.5	564.9
	Yeonje-gu	1,283.0	5.6	27.0	66.3	1,062.1
	Yeongdo-gu	821.4	-	3.0	107.1	510.9
	Jung-gu	1,252.1	-	2.6	62.9	804.2
	Haeundae-gu	541.0	4.8	8.3	29.2	411.6
**Daegu**	**Total**	957.2	317.9	10.3	40.7	484.8
	Nam-gu	2,155.8	1,155.0	15.3	73.6	759.2
	Dalseo-gu	801.7	267.8	4.1	37.6	399.5
	Dalseong-gun	930.1	281.6	4.9	32.0	552.9
	Dong-gu	788.9	208.2	25.8	51.0	370.5
	Buk-gu	696.0	173.9	3.9	22.6	409.7
	Seo-gu	1,810.6	530.0	17.1	35.5	1,054.7
	Suseong-gu	666.4	216.9	10.5	49.3	310.8
	Jung-gu	1,482.3	440.3	12.1	45.2	830.9

**Incheon**	**Total**	1,184.1	2.1	24.6	90.5	949.3
	Ganghwa-gun	992.6	-	29.4	55.5	816.3
						continued
	Gyeyang-gu	1,171.5	2.0	31.8	91.0	943.7
	Namdong-gu	981.8	1.4	20.1	100.5	753.5
	Dong-gu	1,580.8	13.0	57.7	128.3	1,281.4
	Nam-gu	1,330.0	2.0	24.6	57.6	1,138.7
	Bupyeong-gu	1,282.8	2.1	24.1	127.0	999.2
	Seo-gu	1,119.4	0.9	28.2	93.0	874.4
	Yeonsu-gu	1,229.7	3.9	13.1	75.4	1,005.5
	Jung-gu	1,406.4	0.9	30.4	64.2	1,150.3
**Gwangju**	**Total**	530.9	1.3	22.4	69.6	329.4
	Gwangsan-gu	624.3	-	9.9	94.2	419.0
	Nam-gu	449.8	1.7	17.2	45.3	296.2
	Dong-gu	589.4	6.8	32.8	73.5	337.1
	Buk-gu	454.6	1.1	37.3	56.2	269.3
	Seo-gu	558.7	0.8	16.9	73.2	322.7
**Daejeon**	**Total**	777.5	2.4	18.9	35.9	598.3
	Daedeok-gu	685.4	0.7	13.5	38.4	508.8
	Dong-gu	754.3	0.5	20.2	34.6	605.5
	Seo-gu	702.9	3.1	14.9	26.4	541.7
	Yuseong-gu	810.9	5.0	19.1	44.3	620.6
	Jung-gu	968.3	0.5	29.2	42.3	738.4
**Ulsan**	**Total**	584.3	3.1	8.6	61.9	340.5
	Nam-gu	706.3	4.5	10.9	114.4	374.6
	Dong-gu	609.6	4.0	9.5	53.0	403.8
	Buk-gu	548.6	2.9	5.3	39.4	288.7
	Ulju-gun	333.6	0.5	4.4	24.7	192.0
	Jung-gu	673.5	3.3	12.2	49.8	444.6
**Sejong**	**Sejong-si**	558.2	15.4	6.4	23.7	397.2
