# Supplementary material for: Effect of socioeconomic disparities on the risk of COVID-19 in 8 metropolitan cities in the Korea: a community-based study
Source: Epidemiol Health. 2022 Nov 15;44:e2022107. doi: 10.4178/epih.e2022107 (PMC10185970; doi:10.4178/epih.e2022107)
Supplement: Supplementary Material 4. — Relative risk of COVID-19 fatality per interquartile range increment of the level of area deprivation indices [file epih-44-e2022107-Supplementary-4.pdf]

Supplementary materials

Supplementary Material 4. Relative risk of COVID-19 fatality per interquartile range increment of the level of area deprivation indices

Area deprivation index	Model 1		Model 2		Model 3	
	**RR**[†]	**95% CI**	**RR**[†]	**95% CI**	**RR**[†]	**95% CI**
Composite deprivation index	1.37	(1.05–1.77)*	1.42	(1.11–1.80)*	1.45	(1.11–1.80)*
Economic deprivation index	1.23	(0.94–1.60)	1.29	(0.90–1.85)	1.34	(0.90–1.86)
Social deprivation index	1.34	(0.99–1.78)	1.39	(1.00–1.84)*	1.42	(1.06–1.85)*
Deprivation index of factors related to mortality	1.32	(1.03–1.68)*	1.34	(1.09–1.65)*	1.40	(1.09–1.66)*

RR, relative risk; CI, confidence interval. *P-value <0.05. Model 1: crude model; model 2: adjusted with the level of standardized prevalence of hypertension; and model 3: model 2 + adjusted with the standardized prevalence of diabetes.
