# Supplementary material for: Effect of socioeconomic disparities on the risk of COVID-19 in 8 metropolitan cities in the Korea: a community-based study
Source: Epidemiol Health. 2022 Nov 15;44:e2022107. doi: 10.4178/epih.e2022107 (PMC10185970; doi:10.4178/epih.e2022107)
Supplement: Supplementary Material 5. — Relative risk of COVID-19 incidence per interquartile range increment of the standardized prevalence of diabetes [file epih-44-e2022107-Supplementary-5.pdf]

Supplementary materials

Supplementary Material 5. Relative risk of COVID-19 incidence per interquartile range increment of the standardized prevalence of diabetes

Index	Model 1		Model 2		Model 3	
	**RR†**	**95% CI**	**RR†**	**95% CI**	**RR†**	**95% CI**
Standardized prevalence of diabetes (%)	1.04	(1.00–1.08)	1.05	(1.00–1.10)*	1.09	(1.04–1.14)*

RR, relative risk; CI, confidence interval. **P*-value <0.05. RR, Model 1: crude model; model 2: adjusted for composite deprivation index; and model 3: model 2 + adjusted for the standardized prevalence of hypertension.
