# Supplementary material for: Effect of socioeconomic disparities on the risk of COVID-19 in 8 metropolitan cities in the Korea: a community-based study
Source: Epidemiol Health. 2022 Nov 15;44:e2022107. doi: 10.4178/epih.e2022107 (PMC10185970; doi:10.4178/epih.e2022107)

Supplementary materials

Supplementary Material 7. Association between four quantiles for composite deprivation index and regional vaccination rate of SARS-CoV-2 in (A) all and (B) ≥75 years old population as the number of vaccination on November 1, 2021. †Vaccination rate was calculated as the number of SARS-CoV-2 vaccination completed subjects divided by the number of participants to SARS-CoV-2 vaccination.

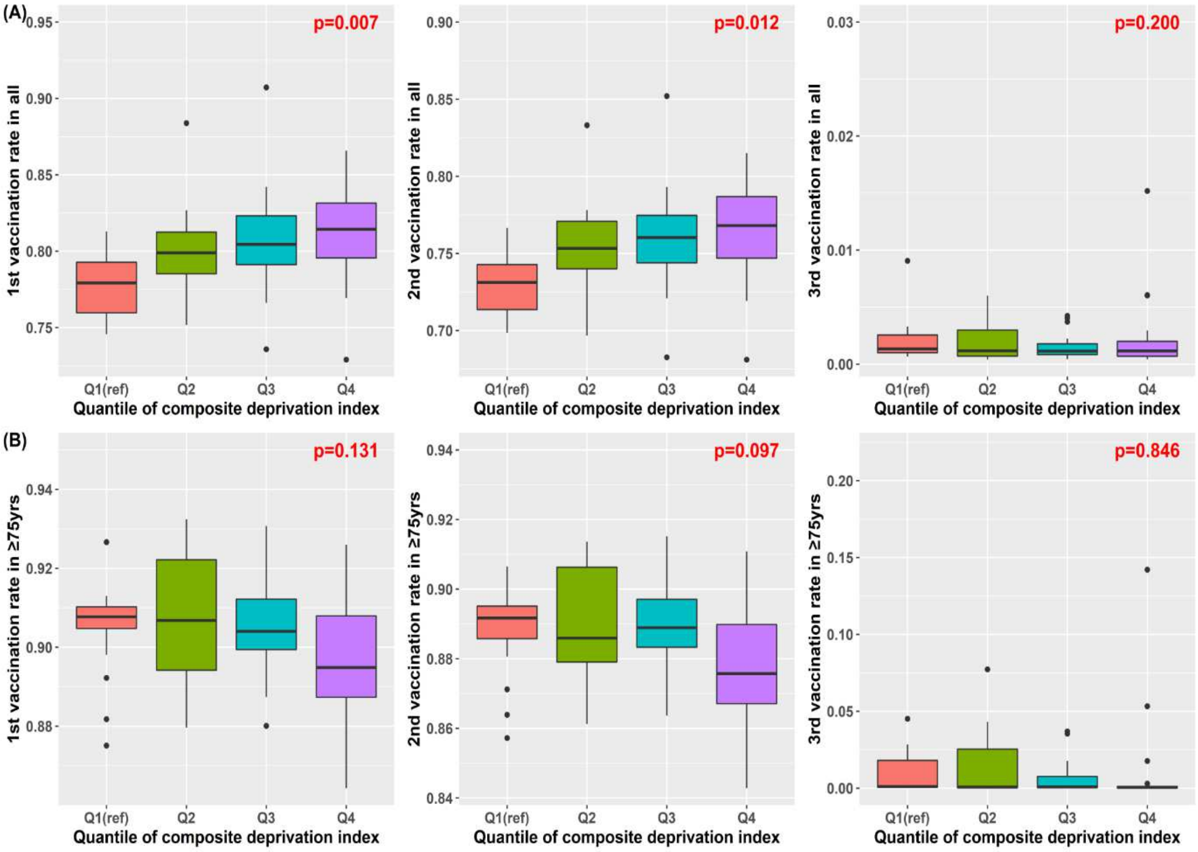

Supplement: Supplementary Material 7. — Association between four quantiles for composite deprivation index and regional vaccination rate of SARS-CoV-2 in (A) all and (B) ≥75 years old population as the number of vaccination on November 1, 2021. †Vaccination rate was calculated as the number of SARS-CoV-2 vaccination completed subjects divided by the number of participants to SARS-CoV-2 vaccination. [file epih-44-e2022107-Supplementary-7.pdf]
